# Bioethical Concerns During the COVID-19 Pandemic: What Did Healthcare Ethics Committees and Institutions State in Spain?

**DOI:** 10.3389/fpubh.2021.737755

**Published:** 2021-10-13

**Authors:** Javier Ruiz-Hornillos, Pilar Hernández Suárez, Juana María Marín Martínez, Íñigo de Miguel Beriain, María Auxiliadora Nieves Vázquez, Marta Albert, María Herrera Abián, Pedro A. Pacheco-Martínez, Victoria Trasmontes, Encarna Guillén-Navarro

**Affiliations:** ^1^Departamento de Bioética Clínica, Hospital Universitario Infanta Elena, Madrid, Spain; ^2^Instituto de Investigación Sanitaria, Fundación Jiménez Díaz (IIS-FJD), Madrid, Spain; ^3^Facultad de Medicina de la Universidad Francisco de Vitoria, Pozuelo de Alarcón, Madrid, Spain; ^4^Comité de Ética Asistencial, Comité de Ética de Investigación con Medicamentos, Hospital Universitario 12 de Octubre, Madrid, Spain; ^5^Servicio de Urgencias del Hospital Clínico Universitario Virgen de la Arrixaca, Murcia, Spain; ^6^Presidente Comité de Ética del Área i del Servicio Murciano de Salud, Murcia, Spain; ^7^Consejo Asesor Regional de Ética Asistencial de Murcia (CAREA), Murcia, Spain; ^8^Facultad de Derecho, Universidad del País Vasco, EuskalHerriko Uniberstiatea, Vizcaya, Spain; ^9^Comité de Ética Asistencial Fundación Jiménez Díaz, Madrid, Spain; ^10^Universidad Rey Juan Carlos, Madrid, Spain; ^11^Unidad de Cuidados Intensivos, Hospital 12 de Octubre, Madrid, Spain; ^12^Sección de Genética Médica, S. Pediatría, Hospital Clínico Universitario Virgen de la Arrixaca, IMIB-Arrixaca, Universidad de Murcia, CIBERER-ISCIII, Murcia, Spain; ^13^Comité de Bioética de España, Madird, Spain

**Keywords:** equity, morbidity, mortality, prioritization, triage, healthcare ethics committees, COVID-19

## Abstract

**Objectives:** Each new wave of the COVID-19 pandemic invites the possible obligation to prioritize individuals' access to vital resources, and thereby leads to unresolved and important bioethical concerns. Governments have to make decisions to protect access to the health system with equity. The prioritization criteria during a pandemic are both a clinical and legal-administrative decision with ethical repercussion. We aim to analyse the prioritization protocols used in Spain during the pandemic which, in many cases, have not been updated.

**Method:** We carried out a narrative review of 27 protocols of prioritization proposed by healthcare ethics committees, scientific societies and institutions in Spain for this study. The review evaluated shared aspects and unique differences and proffered a bioethical reflection.

**Results:** The research questions explored patient prioritization, the criteria applied and the relative weight assigned to each criterion. There was a need to use several indicators, being morbidity and mortality scales the most commonly used, followed by facets pertaining to disease severity and functional status. Although age was initially considered in some protocols, it cannot be the sole criterion used when assigning care resources.

**Conclusions:** In COVID-19 pandemic there is a need for a unified set of criteria that guarantees equity and transparency in decision-making processes. Establishing treatment indications is not the aim of such criteria, but instead prioritizing access to care resources. In protocols of prioritization, the principle of efficiency must vary according to the principle of equity and the criteria used to guarantee such equity.

## Introduction

The COVID-19 pandemic has posed many ethical questions to healthcare professionals worldwide (1–3). One concern, in particular, has arisen due to the shortage of human and material resources within an epidemiologic setting. Indeed, as a result of such a circumstance and despite the efforts undertaken, global health systems have been pushed to their limits. The shortfall—whether temporary or for an extended time—has needed prioritization criteria for accessing to such resources.

Each new wave of the COVID-19 pandemic invites the possible obligation to prioritize individuals' access to vital resources everywhere, and for which some hospitals and institutions have drafted documents that should be analyzed and re-evaluated continually.

Governments have to make decisions to protect access to the health system with equity (4). The prioritization criteria during a pandemic are both a clinical and legal-administrative decision with ethical repercussion. The criteria proposed by European scientific societies differ in some aspects from the recommendations of bioethics committees (5).

Prioritization policies could differ depending on the health system of each country. Most of them emphasized the need to save the greatest number of lives, but they had different approaches on how to achieve it, different clinical criteria to use and ethical principles to defend (6). In Spain, each attempt tried to give an answer to these questions, the scientific societies, the healthcare ethics committees (HECs), so a retrospective analysis of all of them is necessary, providing a bioethical approach. This preliminary work can be the basis to compare priorization policies in other countries, since it is conceivable that in a few years there will be corresponding documents at EU level.

The pandemic in Spain had a considerable impact, so that during the first wave there were more than 2,000 daily hospitalizations for more than 2 weeks, becoming the eighth country with the highest number of cases and a mortality rate that reached being the fifth in the world (60.7/100,000), which meant an overload of the health system (7, 8). In these circumstances, some hospitals, HECs, and institutions were forced to develop documents in which the prioritization of people's access to vital resources was recommended. These documents generated in a situation of exceptionality are still in place in some centers, so a reevaluation and exhaustive analysis of them is necessary to be able to update them to current circumstances.

The pandemic may have revealed the scarce bioethical resources available in our health systems (9). Our aim is to analyse the prioritization protocols used in Spain during the COVID-19 pandemic. Yet, as the current set of prioritization criteria continues presenting several, difficult-to-respond matters, perhaps it is time to reflect on what was proposed and include an ethical vision when considering efficient resource management (10).

## Methods

In an attempt to provide such clarification, we carried out a narrative review of the protocols and documents prepared in Spain by different HECs, scientific societies, and other institutions that have responded to various ethical matters related to the pandemic and scarcity of resources. The search was diversified into documents elaborating by HECs, Hospitals, Universities, Scientific Societies, Regional Institutions and Nacional Institutions, We collected public documents those available on the intranet of these institutions and in hospitals' HEC network, as well as those internal documents sent directly to researchers from this project. The documents were analyzed by the authors, experts in bioethics. We reviewed 85 documents, discarding 58. The main exclusion criterion used was the absence of the prioritization issue in the documents analyzed. 27 documents were selected for a final analysis (11). Fifteen of the documents had been prepared by HECs 3 by Regional Institutions, three by Scientific Societies, three by Hospitals, two by Nacional Institutions, and one by a University. The data to be extracted from the documents were agreed in order to evaluate the answers given to the dilemmas raised by the researchers. So the questions were: Who decides which criteria to use to prioritize individuals?, What should be prioritized: lives / years of life / quality of life?, What criteria should be used?, Is it enough to recommend some scales or is it convenient to prioritize some criteria and set “cut-off points?,” Can an age limit be set for resource access?, Should the use of resources be limited to patients with disabilities?, Should patients with COVID-19 be given higher priority than those without COVID-19?, Is it ethical to prioritize by order of arrival?, Can healthcare professionals be prioritized?, Who makes decisions for each patient? What is the role of HECs in establishing resources access?, Is a discussion on legal regulations necessary to prioritize resources? The answers to these questions were evaluated and. a critical assessment was made from the ethical and/or administrative legal point of view. Below the results obtained for each question are described, with the common points and differences. The summary of the results is presented in Table 1.

**Table 1:** Main data of the analyzed documents.

	**Publication date**	**Scope of action**	**Objective in *triage***.	**How many criteria were used?**	**Were fundtional situation criteria used? (Which?)**	**Were severity criteria used? (Which?)**	**Were Prognostic criteria used? (Which?)**	**Were age criterion used?**	**Was the age limit for access to resources specified?**	**Were quality of life criteria used?**	**Was it prioritized in order of arrival?**	**Were some criteria prioritized** **over others?**	**Were non-covid patients taken into account?**	**Was health care prioritized?**
1. DBC del H.U. Infanta Elena (Valdemoro, Madrid)	10-3-20	EC	N°L	4	Yes (Barthel)	Yes (SOFA)	Yes (Charlson)	Yes	No *R(a)	No	No	Yes (F > S > P)	YES	No
2. CEAS H.U. La PriNDesa (Madrid)	13-3-20	EC	N°L > N°Y	5	Yes (NS)	Yes (Apache)	Yes (NS)	Yes	yes, <80 años	No	Yes	No	Yes	No
3. CEAS H. Clínico San Carlos. (Madrid)	16-3-20	EC	N°L	5	Yes (NS)	Yes (SOFA y Apache)	Yes (NS)	Yes	yes, <80 años	Yes (NS)	No	No	Yes	No
4. CEAS HUF Alcorcón. (Madrid)	16-3-20	EC	N°L > N°Y	3	Yes (Barthel)	No	Yes (Charlson)	Yes	No	No	No	Yes (P > F > A)	No	No
5. CEAS H. Severo Ochoa. (Madrid)	17-3-20	EC	N°L	4	No	Yes (SOFA y Berlin)	Yes (Charlson)	Yes	No	No	R	No	Yes	No
6. CEAS Getafe. (Madrid)	18-3-20	EC	N°L > N°Y	4	Yes (Barthel)	Yes (NS)	Yes (Charlson y Profund)	Yes	No * R(a)	No	No	Yes (S>P>A)	Yes	No
7. Grupo bioética SEMICYUC	20-3-20	SS	N°L > AC	4	Yes (NS)	Yes (SOFA)	Yes (NECPAL)	Yes	yes, <80 años	Yes (NS)	R	Yes (S > P > A)	Yes	No
8. CEAS H. U. La Paz. (Madrid)	22-3-20	EC	N°L	2	Yes (NS)	No	Yes (NS)	Yes	No * R(a)	No	No	No	No	No
9. CEAS H.U. Cruces. (Barakaldo, Bizcaia)	23-3-20	EC	NS	2	No	Yes (NS)	No	No	No	Yes (NS)	No	No	No	No
10. UCI Osakidetza. País Vasco	23-3-20	RI	N°L > N°Y	4	Yes (Barthel)	Yes (SOFA)	Yes (Por patologías)	Yes	yes, <80 años	No	No	Yes (S > P > A)	No	No
11. Comité de Bioética de España	23-3-20	NI	N°L	NS	No	No	No	Yes	No * R(a)	No	R	No	Yes	Yes
12. CEAS C.H.U. de Badajoz.	24-3-20	EC	N°L	3	Yes (NS)	Yes (NS)	Yes (NS)	Yes	No * R(a)	No	No	Yes (F/P)	Yes	No
13. Comité de Bioética de Cataluña	24-3-20	RI	N°L > N°Y > QL	3	Yes (NS)	Yes (SOFA)	Yes (NS)	Yes	No	Yes (NS)	No	Yes(S = P = A)	No	No
14. CEAS H.G.U. José María Morales Meseguer (Murcia)	24-3-20	EC	N°L	5	Yes (Barthel)	Yes (SOFA)	Yes (Profund o NECPAL)	No	No	Yes (ECOG)	No	Yes (S > P/Q)	No	No
15. CEAS H. U. Clinic Barcelona.	26-3-20	EC	NS	4	Yes (NS)	Yes (SOFA)	Yes (MACA o PCC)	Yes	yes, <80 años	Yes (NS)	R	Yes (F > P > A)	No	No
16. Observatorio de Bioética Y Derecho de la Universidad de Barcelona.	30-3-20	RI	N°L > QL	5	Yes (Barthel)	Yes (SOFA o Apache)	Yes (MACA o PCC)	Yes	yes, <80 años	Yes (NS)	No	No	No	No
17. Comisión Asesora de Bioética Del PriNDipado de Asturias CABEPA.	31-3-20	EC	N°L	NS	Yes (NS)	No	No	No	No * R(a)	No	R	Yes (F)	Yes	No
18. Ministerio de Sanidad.	3-4-20	NI	N°L	NS	No	No	No	No	No * R(a)	No	R	No	Yes	No
19. H. Ernest Lluch de Calatayud (Zaragoza)	16-4-20	H	NS	2	No	Yes (NS)	Yes (NS)	No	No	No	No	No	No	No
20. Cátedra de Cuidados Paliativos, U. de Vic (UVIC-UCC),	abr-20	U	NS	2	Yes (Frágil-VIG o CFS)	No	Yes (MACA, PCC o NECPAL)	No	No * R(a)	No	No	No	No	No
21. Sociedad de Geriatría y Gerontología	4-5-20	SS	NS	3	Yes (NS)	Yes (NS)	Yes (NS)	No	No	No	No	No	No	No
22. Sociedad Cardiología Geriátrica	11-6-20	SS	N°L	2	Yes (NS)	No	Yes (NS)	No	No * R(a)	No	No	No	No	No
23. CEAS H.U Donostia	ND	EC	N°L > N°Y	3	No	Yes (NS)	Yes (NS)	Yes	No * R(a)	No	R	No	Yes	Yes
24. CEAS H.M. Hospitales. Triaje	ND	EC	N°L	3	No	Yes (NS)	Yes (NS)	Yes	No * R(a)	No	No	No	Yes	No
25. H. U. Puerta de Hierro	ND	H	N°L	3	Yes (NS)	Yes (SOFA)	Yes (NS)	Yes	No * R(a)	No	No	Yes (F > P)	No	No
26. CEAS de Segovia.	ND	EC	N°L > QL	3	No	Yes (NS)	Yes (NS)	Yes	No * R(a)	No	R	No	No	No
27. Medicina Intensiva del H. Vall d'Hebron (Barcelona)	ND	H	NS	5	Yes (NS)	Yes (NS)	Yes (NS)	Yes	yes, <80 años	Yes (NS)	No	Yes (A > P)	No	No

ND, No data; EC, Ethics Committees; H, Hospital; RI, Regional Institution; NI, Nacional Institution; SS, Scientific Society; U, Universidad; N°L, To save as many lives as possible; N°Y, To maximize the number of years of life saved or increase the chances of living longer; QL, To prioritize the quality of life; NS, Not specified; *R(a), Explicit refusal to use age as the sole criterion to limit access to a resource: R, Rejects it as a criterion; F, Fundtional; S, Severity; P, Pronostic: A, Age; Q, quality of life.

## Results

### Q1. Who Decides Which Criteria to Use to Prioritize Individuals?

To know how these decisions were made in the pandemic, it is necessary to remember the sequence of events.The first case of COVID-19 in Spain was on 31 January 2020 (7). On 1 March, 83 cases had been confirmed, and the spread of the virus became widespread throughout the national territory. On 10 March, more than 1,500 were already infected, whilst on 11 March, 2020, the World Health Organization (WHO) considered that COVID-19 had to be characterized as a pandemic. On 12 March, RD 6/2020 was approved, empowering the State Health Administration to act in the event of a shortage of medicines, health products, or any product necessary to protect health (12). However, it was not until April 2 when the Ministry of Health issued its first report on ethical aspects in pandemic situations (13). At this time, there were more than 120,000 infected and 11,700 deaths. The absence of centralized guidelines had, however, led many HECs and institutions to prepare their own documents with recommendations on prioritization prior. The first of these documents was published on 10 March, 2020, by the Department of Bioethics at Infanta Elena University Hospital (14). Since then, and until the dissemination of the Ministry of Health's recommendations, on 2 April, 2020, another 16 documents were identified including the critical document from the Bioethics Committee of Spain, on March 25, about ethical aspects of priorization in health resources (15).

### Q2. What Should Be Prioritized: Lives/Years of Life/Quality of Life?

Another concern issue is to determine the objective in prioritization, as several approaches are possible (16–18):

- To save as many lives as possible.
- To maximize the number of years of life saved or increase the chances of living longer.
- To prioritize the quality of life, i.e., disability-free survival over isolated survival (to maximize the number of QALYs, quality-adjusted life years).

When analyzing the documents, 12 of 27 (44.4%) stated that the objective was to save the greatest number of lives. Another six (22.2%) proposed a combination of the first two points, and four (14.8%) proposed integrating quality of life in the objectives. The remaining documents (18.5%) did not explicitly comment on this objective.

### Q3.What Criteria Should Be Used?

Most of the documents reviewed (88.9%) recommended certain criteria to take into account during prioritization (Table 1). All recommended to use a combination of several criteria, between 2 and 5, with a median of four.

The criteria used can be divided into:

- Criteria related to the clinical situation or severity of the patient, collected in 20 (74.1%) documents. The SOFA scale was the most used, followed by APACHE.
- Prognostic criteria for morbidity and mortality in 23 (85.2%) documents. The most recommended criteria included the Charlson scale, MACA, PPC, PROFUND and NECPAL. The majority did not, however, specify the scales to be used (51.8%).
- Age criterion, collected in 19 (70.4%) documents, which is analyzed later.
- Functional situation of the patient (recommended in 19 (70.4%) documents), with the Barthel scale being the most used.
- The patient's quality of life of the patient was recommended in eight (29.6%) documents, amongst which only one specified the ECOG scale.

### Q4. Is It Enough to Recommend Some Scales or Is It Convenient to Prioritize Some Criteria and Set “Cut-Off Points”?

The levels of recommendation varied: in some cases, the clinical or functional situation of the patients was recommended to be taken into account, whilst in other instances, the use of certain scales, such as the aforementioned Brathel, SOFA, etc., was proposed. Only some (44.4%) ranked some criteria: six prioritized severity and prognosis criteria, whilst five the patient's functional situation.

### Q5. Can Age Limit Be Set for Resource Access?

The document presented by the Ministry of Health in 2 April 2020 (13) shows that the age criterion cannot be used to deny or limit health care and the use of certain measures. It maintains “the absolute ban on criteria based on discrimination for any reason to prioritize patients within those contexts. excluding patients from access to certain resources (e.g., applying said limitation to anyone aged >80 years) is contrary, by discrimination, to the fundamentals dictated by our rule of law” (Article 14 of the Spanish Constitution).

Previously, the Bioethics Committee of Spain (15) had already established that the age criterion could not be used to deny or limit health care and the use of certain life support measures.

However, the age criterion was included as a priority criterion in six of the protocols that were published previously to the Ministry of Health document, namely limiting access to intensive care to those aged >80 years. The Semicyuc document (19) recommended that patients with similar characteristics prioritize the person with the longest QALY. In the remaining protocols, age was included as one more criterion to consider when prioritizing, e.g., in the Charlson scale, age effectively influences the clinical prognosis. Non-etheless, 12 (44.4%) documents reflect an explicit refusal to use age as the sole criterion to limit access to a resource.

### Q6. Should the Use of Resources Be Limited to Patients With Disabilities?

The presence of the recommendation to prioritize disability-free survival over isolated survival in the Semicyuc protocol (19) of 23 March motivated the Directorate General for Disability Policies of the Ministry of Social Rights and Agenda 2,030 to consult with the Bioethics Committee of Spain. The latter indicated in its report (15) that the criteria used must respect the dignity of the person, as well as the equity and protection against those vulnerable; The document published by the Ministry of Health (13) also recommends avoiding discrimination based on disability, explaining that the only valid reasons for prioritization would be the patient's clinical situation and objective survival expectations.

### Q7. Should Patients With COVID-19 Be Given Higher Priority Than Those Without COVID-19?

In this sense, 11 (40.7%) documents do reveal the need to allocate resources to patients with non-COVID-19 pathologies to avoid discrimination. Some documents explain that prioritization criteria must be the same for all pathologies, even prioritizing patients without COVID-19 due to the known resource efficiency in these cases.

### Q8. Is It Ethical to Prioritize by Order of Arrival?

The criterion “first to arrive, first to be admitted”, was considered as a possibility in only one case; eight (28.6%) explicitly rejected it. Several documents also criticized this principle (20), since the use of a resource may imply that it is denied to another person who could benefit more or could unfairly be detrimental to those who become ill later (3). In this sense, the Bioethics Committee of Spain clarifies in its document that this criterion would not respect the principles of equality and justice (15).

### Q9. Can Healthcare Professionals Be Prioritized?

None of the documents raised the possibility of prioritizing care to healthcare professionals with respect to resource allocation. The document presented by the Bioethics Committee of Spain (15) does address it, suggesting it could be ethically acceptable per the principle of justice to prioritize individuals who have placed their health at a greater risk. Similarly, by virtue of the ethical principle of reciprocity, society must support people who accept a disproportionate burden or risk to protect the public good.

### Q10. Who Makes Decisions for Each Patient? What Is the Role of HECs in Establishing Resources?

Caring for patients with and without COVID-19 raises the question of who should make the decision regarding prioritization. Should it be the medical team, critical services, ethics committees or a group of experts created ad hoc? On one hand, decision-making by people outside the supervising medical team, be it the HECs or a group of experts, could improve impartiality and reduce emotional overload for the aforementioned team. On the other hand, HECs have been suggested as not being designed for this type of decision-making processes. It seems clearer then, that the decision be based on a clinical assessment made by the supervising medical team that personalizes protocols to a patient's specific situation.

In this respect, the document presented by the Ministry of Health is unambiguous in that decisions will be made by the supervising medical team, so that a third party is unsuitable to impose criteria unless such party be involved in the patient's care. However, the Ministry also recommends the benefit of requesting or receiving guidance from reference HECs when made possible by time availability or from other physicians with more experience.

## Discussion

Q1. In an attempt to answer this question, we should consider that prioritizing people does not merely comprise establishing scientific-medical criteria to select patients who will benefit the most from a certain resource. Prioritization, in fact, alludes to restricting or suspending a constitutional right to health protection due to the scarcity of resources. In other occasions, however, such prioritization would not be carried out otherwise, given that adaptation of therapeutic efforts for the benefit of the individual and not due to a lack of resources.

Establishing prioritization criteria is both a clinical and legal-administrative decision, for which the report by the Bioethics Committee of Spain (15) urged the Ministry of Health to lead and coordinate the development of common, unique criteria for the entire national territory (13, 21). During the pandemic there can be collisions of rights and governments have to make decisions to guarantee access for all people to available health resources (4). Neither professionals nor scientific societies set health priorities with respect to the application of new treatments, vaccinations, etc. The health authority should set prioritization criteria; the hospital management bodies should agree on a protocol, as it concerns applying these criteria; and the medical team should make a decision that is in accordance with a patient's specific situation. In this way, the healthcare provider will prudently apply criteria that have previously been adopted by the authority, whilst considering the context and particularities of a patient.

Unfortunately, this procedure was not that followed during the first wave of the pandemic. But from this learning, the prioritization of vaccines was carried out with a more logical sequence in Spain. The Ministry of Health proposed a technical committee made up of lawyers, scientists and ethicists and they established recommendations to prioritize vaccines in people with a higher risk of serious disease, so as to guarantee equity at the national level. The criteria were communicated transparently and assumed by all regional institutions and hospitals. In addition, they were periodically reviewed to adapt to events (22).

Q2. This is an essential question because its consequences are decisive in establishing prioritization criteria. Choosing the first criterion considers the equal value of all human life. It would not, therefore, be possible to make distinctions between individuals on other factors except for the probability of survival. On the other hand, adopting the second criterion would make it possible to deny older people access to resources if other younger people or people with underlying pathologies that decrease life expectancy were in need of such. If the third criterion receives priority, access to health resources could be denied to people with disabilities, chronic diseases, dependents, etc.

The document presented by the Ministry of Health (13) states that the objective should be the maximum benefit conferred when saving possible lives, a criterion recommended by the WHO (23) and the only one that conforms to the Spanish Constitution.

Q3. We were facing a disease, in which evolution and treatment response were unknown, or at least, not as much as in other pathologies. It was not often possible to know which patients did or did not benefit more from a resource. Compounding this was the possibility that there were no resources for all those who could benefit from such.

The use of several criteria in most of the protocolsmay indicate that no value is in itself sufficient to determine which patients should receive scarce resources. It further underlies an apparent consideration by all documents, namely the use of multiple values was necessary to make a fair decision on resource allocation and it was, therefore, adaptable to the context of the patient (3).

Q4. The first approach that does not establish clear recommendations incurs the risk of being ambiguous; however, if the criteria established is too narrow, the professional loses the flexibility needed when evaluating each patient. Retrospective studies assessing results obtained with the different scales are warranted to establish more precise recommendations.

Q5. The introduction of the age criterion may have to do with a possible erroneous approach to prioritization objectives. If it is intended exclusively to maximize the use of healthcare resources, that objective will lead to the adoption of criteria that are discriminatory from a constitutional point of view. A priority objective could be to guarantee equitable access to the constitutional right to health protection, as established by the Oviedo Convention and the Universal Declaration of Bioethics and Human Rights. Efficiency alone should, therefore, not be taken into account without the integration of the principle of equity (21).

Q6. Prejudice against people with disabilities should be avoided. Doing otherwise would be incompatible with the International Convention on the Rights of Persons with Disabilities, which was ratified by Spain in 2008.

Q7. There is a risk of allocating most resources to patients with COVID-19 during the pandemic at the expense of patients with other pathologies. Similarly, on some occasions, the effectiveness, and efficiency whilst using resources in the former group may not be as clearly defined as in patients with other intensive pathologies such as stroke, acute myocardial infarction, multiple trauma or in cases of oncological surgeries. Therefore, resource distribution should be fair, and proportioned in such scenarios.

Q10. The criterion of adopting prioritization decisions through healthcare professional groups has also been proposed in other countries. For example, a document prepared by the German Society for Intensive Care (24) suggests the intervention of two intensive care physicians, a representative from the nursing staff and a person with other training, such as clinical ethics.

### Q11. Is a Discussion on Legal Regulations Necessary to Prioritize Resources?

As indicated at the beginning of the document, common prioritization criteria should be established throughout the national territory by those who have the powers to complete such actions, which in this case, is the Ministry of Health. Within the inter-territorial council, it must urge the creation of a task force and the elaboration of a document that provides a framework for decision-making processes in line with constitutional order and recommendations generated by national and international bioethics committees within the last, few months. Such an action has been undertaken in the case of prioritization of vaccine administration (17).

## Conclusions

Prioritizing people's access to vital resources has generated several concerns that have led various HECs and institutions to try to resolve them differently This has been reflected in the great heterogeneity of the different prioritization documents. Unifying criteria would be necessary to guarantee fairness and transparency in decision-making processes. The report by the Ministry of Health on the ethical aspects in pandemic situations can be a model framework; however, a document at a national level that specifies certain aspects is necessary. It is not a matter of whether to establish an indication or not for a resource; rather, it is about the prioritization of access to resources. The principle of efficiency and prioritization criteria pertaining to such principle (which was the first response given from the HECs, in general) must be modulated by the principle of equity and the introduction of prioritization criteria that guarantee the prior. An update and adaptation of initial protocols to the healthcare practices of each hospital are necessary. In this respect, HECs have an important role in the participation of multidisciplinary teams that help professionals who request such guidance during decision-making processes.

## Author Contributions

JR-H, ÍM, MH, and PP-M: conceived and designed the revision of the protocols. PH, JR-H, JM, and MN: reviewed the protocols. JR-H and EG-N: conceived the manuscript. JR-H: made the initial version of the manuscript. All authors the signatories contributed to the analysis and interpretation of the data, made a critical review of the article with important intellectual contributions to it, and approved the final version for approval.

## Funding

This study has received public funding from the INSTITUTO DE SALUD CARLOS III, Ministerio de Ciencia e Innovación de España (COV20/00181).

## Conflict of Interest

The authors declare that they have no conflicts of interest in the preparation of this manuscript.

## Publisher's Note

All claims expressed in this article are solely those of the authors and do not necessarily represent those of their affiliated organizations, or those of the publisher, the editors and the reviewers. Any product that may be evaluated in this article, or claim that may be made by its manufacturer, is not guaranteed or endorsed by the publisher.
